# Transient and Sustained Bacterial Adaptation following Repeated Sublethal Exposure to Microbicides and a Novel Human Antimicrobial Peptide

**DOI:** 10.1128/AAC.03364-14

**Published:** 2014-10

**Authors:** Sarah Forbes, Curtis B. Dobson, Gavin J. Humphreys, Andrew J. McBain

**Affiliations:** a Manchester Pharmacy School, The University of Manchester, Manchester, United Kingdom; b Faculty of Life Sciences, The University of Manchester, Manchester, United Kingdom

## Abstract

Microbicides (biocides) play an important role in the prevention and treatment of infections. While there is currently little evidence for in-use treatment failures attributable to acquired reductions in microbicide susceptibility, the susceptibility of some bacteria can be reduced by sublethal laboratory exposure to certain agents. In this investigation, a range of environmental bacterial isolates (11 genera, 18 species) were repeatedly exposed to four microbicides (cetrimide, chlorhexidine, polyhexamethylene biguanide [PHMB], and triclosan) and a cationic apolipoprotein E-derived antimicrobial peptide (apoEdpL-W) using a previously validated exposure system. Susceptibilities (MICs and minimum bactericidal concentrations [MBCs]) were determined before and after 10 passages (P10) in the presence of an antimicrobial and then after a further 10 passages without an antimicrobial to determine the stability of any adaptations. Bacteria exhibiting >4-fold increases in MBCs were further examined for alterations in biofilm-forming ability. Following microbicide exposure, ≥4-fold decreases in susceptibility (MIC or MBC) occurred for cetrimide (5/18 bacteria), apoEdpL-W (7/18), chlorhexidine (8/18), PHMB (8/18), and triclosan (11/18). Of the 34 ≥4-fold increases in the MICs, 15 were fully reversible, 13 were partially reversible, and 6 were nonreversible. Of the 26 ≥4-fold increases in the MBCs, 7 were fully reversible, 14 were partially reversible, and 5 were nonreversible. Significant decreases in biofilm formation in P10 strains occurred for apoEdpL-W (1/18 bacteria), chlorhexidine (1/18), and triclosan (2/18), while significant increases occurred for apoEdpL-W (1/18), triclosan (1/18), and chlorhexidine (2/18). These data indicate that the stability of induced changes in microbicide susceptibility varies but may be sustained for some combinations of a bacterium and a microbicide.

## INTRODUCTION

Microbicides have been used for more than a century to control microbial growth in the domiciliary, clinical, and industrial environments (1–4). The modes of action of cationic agents, such as quaternary ammonium compounds (QACs) (e.g., cetrimide) and biguanides (e.g., chlorhexidine and polyhexamethylene biguanide), are believed to rely largely on interactions with the bacterial cell envelope, leading to membrane disruption and the leakage of cytoplasmic components (5). Microbicides may also interact with specific pharmacological targets, such as the enoyl-acyl carrier protein reductase FabI, which is a major target of triclosan (1, 6).

Microbicidal compounds are used for a range of applications, including clinical antisepsis and disinfection (2, 4) and the control of biofouling and contamination in industry (7, 8) and in food production (9, 10), while in the domestic setting, they have been incorporated as hygienic adjuncts into various products, including hand washes (11) and hard surface disinfectants (12). There is also increasing interest in their incorporation into medical devices such as urinary catheters (2) and surgical dressings (4) with the intention of inhibiting bacterial colonization and biofilm formation (13–16).

Despite the demonstrable benefits of microbicides in some applications (2, 17–20), concerns have been raised that their extensive use may select for bacteria with reduced susceptibility (21–23). This could occur through the selection of target site-adapted mutants (6) or reversibly through induced phenotypic adaptation (24). While bacterial insusceptibility to in-use concentrations of microbicides is apparently uncommon, there is some evidence of bacteria surviving an antimicrobial challenge, for instance, in microbicide-containing solutions (25), leading to product contamination-related outbreaks (26). It is, however, important to note when considering such reports that microbicides exhibit a spectrum of activity and that some microorganisms may be nonsusceptible without prior microbicide exposure (27). It is therefore possible that in some cases sublethal microbicide exposure could result in the clonal expansion of preexisting bacterial populations with comparatively lower intrinsic susceptibilities, rather than the clonal selection of resistance (28).

While induced changes in antimicrobial susceptibility have been reported in laboratory studies through the exposure of bacteria to sublethal concentrations of microbicides (24, 29, 30), there are very few reports in the literature that document the stability of such changes or that compare the selective potential of multiple microbicides against a range of taxonomically diverse bacteria. We have therefore assessed the potential changes in susceptibility for multiple bacteria after repeated exposure to the microbicides cetrimide, chlorhexidine, polyhexamethylene biguanide (PHMB), and triclosan and a novel human apolipoprotein E-derived antimicrobial peptide (apoEdpL-W). Additionally, since previous investigations have indicated that microbicide (30) and antibiotic (31) adaptations may result in alterations in biofilm-forming ability, the influence of sublethal microbicide exposure on bacterial biofilm formation was also evaluated.

## MATERIALS AND METHODS

### Bacteria.

Pseudomonas aeruginosa ATCC 9027, Staphylococcus aureus ATCC 6538, and Serratia marcescens ATCC 13880 were obtained from Oxoid (Basingstoke, United Kingdom). Burkholderia cepacia ATCC BAA-245, Escherichia coli ATCC 25922, and Klebsiella pneumoniae ATCC 13883 were obtained from the Leibniz Institute DSMZ-German Collection of Microorganisms and Cell Cultures (Braunschweig, Germany). Micrococcus luteus MRBG 9.25, Staphylococcus caprae MRBG 9.3, Staphylococcus capitis MRBG 9.34, Staphylococcus lugdunensis MRBG 9.36, Staphylococcus warneri MRBG 9.27, Staphylococcus epidermidis MRBG 9.33, and Staphylococcus haemolyticus MRBG 9.35 were previously isolated from the axillae of three healthy male volunteers (23). Bacillus cereus MRBG 4.21, Stenotrophomonas maltophilia MRBG 4.17, and Chryseobacterium indologenes MRBG 4.29 were isolated from a domestic kitchen drain biofilm (28). Enterococcus faecalis WIBG 1.1 and Corynebacterium xerosis WIBG 1.2 were wild-type wound isolates provided by Angela Oates, The University of Manchester.

### Chemical reagents and bacterial growth media.

Triclosan, cetrimide, and chlorhexidine were purchased from Sigma-Aldrich (Dorset, United Kingdom). Vantocil (a 20% aqueous solution of PHMB) was obtained from Arch Chemicals, Inc. (Manchester, United Kingdom). Peptides were purchased from Alta Bioscience (West Midlands, United Kingdom), having been synthesized using 9-fluorenylmethyl carbamate chemistry and purified by high-performance liquid chromatography. Bacteriological media were purchased from Oxoid. All other chemical reagents were purchased from Sigma-Aldrich unless otherwise stated. Bacterial growth media were sterilized at 121°C and 15 lb/in^2^ for 15 min prior to use. All bacteria were cultured on Mueller-Hinton agar (Oxoid) and incubated aerobically at 37°C for 18 h unless stated otherwise.

### Determination of bacterial MICs and MBCs.

The MICs were determined using the microdilution method as described previously (23, 32). Briefly, overnight bacterial cultures were adjusted to an optical density at 600 nm (OD_600_) of 0.8 and diluted 1:100 in Mueller-Hinton broth in a 96-well microtiter plate containing doubling dilutions of the relevant microbicide. All microbicide stock solutions were prepared at 5 times the highest test concentration in water and filter sterilized (0.22 μM). The plates were incubated at 37°C (24 h) with agitation (100 rpm). The MIC was defined as the lowest concentration for which bacterial growth did not occur. Growth was viewed as turbidity (600 nm) in comparison to that of an uninoculated well (negative control) and was detected using a microtiter plate reader (PowerWave XS; BioTek, Bedfordshire, United Kingdom). Aliquots (10 μl) from wells exhibiting no turbidity were transferred to sterile Mueller-Hinton agar and incubated (37°C) for the determination of the minimum bactericidal concentration (MBC). The MBC was defined as the lowest concentration of microbicide at which no growth occurred after 4 days of incubation.

### Biofilm formation assay.

Overnight cultures of test bacteria were adjusted to an OD_600_ of 0.8 then diluted 1:100 in sterile Mueller-Hinton broth. A volume of 150 μl of diluted bacterial inoculum was delivered to each test well of a 96-well microtiter plate. Plates were incubated for 48 h at 37°C and 20 rpm to promote biofilm growth. Wells were washed twice with 250 μl of sterile phosphate-buffered saline (PBS) before 200 μl of 0.5% (wt/vol) crystal violet solution was added to the test wells. Plates were incubated for 30 min at room temperature, and the wells were subsequently washed twice with 250 μl of PBS and left to dry at room temperature for 1 h. The attached crystal violet was solubilized in 250 μl of 95% ethanol per well, and plates were agitated at room temperature at 20 rpm for 1 h. After solubilization, biofilm growth was viewed as the change in OD_600_ relative to that in a sterile negative control (33). Biofilm-bound crystal violet was quantified for P0 and P10 bacteria, and average values were calculated using data from two separate experiments, each with three technical replicates (*n* = 6). Statistical significance was determined using a paired Student's *t* test (*P* < 0.001).

### Exposure of bacteria to sublethal concentrations of microbicides.

A previously validated system (23) was used to generate reproducible ca. 100-fold antimicrobial concentration gradients on Mueller-Hinton agar plates using a spiral plater (Whitley automated spiral plater; Don Whitley Scientific, Shipley, United Kingdom). The initial antimicrobial stock solutions (50 μl) were deposited on the agar surface. Plates were dried for 1 h at room temperature prior to radial deposition of bacterial pure cultures and then incubated (4 days at 37°C) in a static aerobic incubator. After incubation, growth observed at the highest microbicide concentration was aseptically removed and radially streaked onto a fresh plate containing the same antimicrobial concentration gradient. If growth was observed across the whole antimicrobial gradient, a new plate containing a 5× higher stock solution concentration was used (30). This process was repeated until 10 passages had occurred. Bacteria were then passaged a further 10 times in the absence of any antimicrobial (X10). Bacteria at P0, P10, and X10 were archived for subsequent MIC and MBC testing.

## RESULTS

### Cetrimide.

The majority of the test bacteria in this study underwent comparatively minor reductions (≤2-fold) in susceptibility following repeated exposure to cetrimide (Table 1). From those that underwent a ≥4-fold decrease, three of the five strains were staphylococci, with the MICs increasing by in excess of 18-fold for S. haemolyticus. Following the cessation of microbicide exposure, full reversions in the MICs occurred for E. coli, K. pneumoniae, and S. epidermidis, while no reversion in the MICs was apparent for S. haemolyticus and S. lugdunensis. E. coli was the only bacterium to display a ≥4-fold increase in the MBC, which completely reverted when grown in the absence of cetrimide.

**TABLE 1:** MICs and minimum bactericidal concentrations of bacteria before and after treatment with cetrimide^*a*^

Test bacterium	MIC (μg/ml)			MBC (μg/ml)		
	Before exposure	P10	X10	Before exposure	P10	X10
Bacillus cereus	7.3	14.5	7.3	14.5	48.3 (8)	29
Burkholderia cepacia	38.7 (17)	38.6 (8)	29	116	232	232
Chryseobacterium indologenes	12.1 (4)	14.5	14.5	29	29	29
Corynebacterium xerosis	3.6	3.6	3.6	14.5	9.7 (4)	14.5
Enterococcus faecalis	12.1 (4)	14.5	14.5	29	38.7 (16)	58
Escherichia coli	29.17 (8)	**116**	29	116	**464**	116
Klebsiella pneumoniae	29.3 (8)	**116**	29	29	58	58
Micrococcus luteus	14.5	7.3 (33)	14.5	58	19.3 (8)	29
Pseudomonas aeruginosa	232	232	232	464	464	464
Serratia marcescens	24.2 (8)	37.3 (14)	29	37.3 (14)	116	58
Staphylococcus aureus	4.8 (2)	6 (2)	7.3	7.3	14.5	7.3
Staphylococcus capitis	3.6	7.3	7.3	14.5	7.3	7.3
Staphylococcus caprae	0.9	1.8	1.8	14.5	14.5	14.5
Staphylococcus epidermidis	1.8	**7.3**	1.8	3.6	7.3	7.3
Staphylococcus haemolyticus	0.4	**7.3**	**7.3**	14.5	14.5	14.5
Staphylococcus lugdunensis	0.4	**3.6**	**3.6**	29	14.5	29
Staphylococcus warneri	4.8 (2)	6.1 (2)	7.3	193.3	232	116
Stenotrophomonas maltophilia	19.3 (8)	29	29	58	24.2 (8)	58

a Data show the mean MICs and minimum bactericidal concentrations of bacteria before and after microbicide exposure in μg/ml and represent samples taken from two separate experiments each with three technical replicates. For data that varied between replicates, SDs are given in parentheses. Bold type indicates a ≥4-fold change when comparing P0 to P10 and X10 values.

### Chlorhexidine.

Data in Table 2 indicate that ≥4-fold increases in the MICs occurred for B. cepacia, E. faecalis, K. pneumoniae, S. marcescens, S. lugdunensis, and S. maltophilia following 10 passages in the presence of chlorhexidine. E. faecalis demonstrated a complete reversion in the MIC after the cessation of microbicide exposure. Partial reversions (MICs) were observed for B. cepacia and S. marcescens, while K. pneumoniae, S. maltophilia, and S. lugdunensis values failed to revert when passaged in the absence of chlorhexidine. Partial reversions in MBCs were observed in B. cereus, B. cepacia, S. marcescens, and S. aureus, while S. lugdunensis and S. maltophilia did not revert significantly after growth in a microbicide-free environment.

**TABLE 2:** MICs and minimum bactericidal concentrations of bacteria before and after treatment with chlorhexidine^*a*^

Test bacterium	MIC (μg/ml)			MBC (μg/ml)		
	Before exposure	P10	X10	Before exposure	P10	X10
Bacillus cereus	14.5	14.5	14.5	29	**232**	**116**
Burkholderia cepacia	3.6	**29**	7.3	26.6 (6)	**232**	**116**
Chryseobacterium indologenes	7.3	7.3	7.3	7.3	14.5	7.3
Corynebacterium xerosis	3.3 (1)	3.6	3.6	21.8 (8)	14.5	14.5
Enterococcus faecalis	3.6	**24.2 (8)**	3.6	26.6 (6)	58	29
Escherichia coli	6.7 (1)	7.3	7.3	13.3 (3)	29	29
Klebsiella pneumoniae	2.1 (1)	**14.5**	**14.5**	16.3 (5)	58	**116**
Micrococcus luteus	3.6	3.6	3.6	14.5	7.3	14.5
Pseudomonas aeruginosa	7.3	14.5	7.3	14.5	29	14.5
Serratia marcescens	12.1 (4)	**116**	**58**	24.2 (7)	**232**	**116**
Staphylococcus aureus	8.5 (4)	3.6	3.6	13.3 (4)	**58**	29
Staphylococcus capitis	3.6	6 (2)	7.3	14.5	14.5	29
Staphylococcus caprae	3.6	3.6	7.3	29	29	29
Staphylococcus epidermidis	13.3 (3)	9.7 (4)	14.5	33.8 (12)	24.2(8)	29
Staphylococcus haemolyticus	1.4 (0.4)	3 (1)	1.8	4.2(1)	14.5	7.3
Staphylococcus lugdunensis	0.9	**3.6**	**4.8 (2)**	1.7 (0.3)	**48.3 (17)**	**58**
Staphylococcus warneri	29	29	29	58	58	58
Stenotrophomonas maltophilia	4.8 (2)	**29**	**29**	14.5	**58**	**58**

a See Table 1 footnote for explanation of data.

### PHMB.

After 10 passages in the presence of PHMB, ≥4-fold increases in the MICs were observed for C. indologenes, E. faecalis, K. pneumoniae, M. luteus, S. capitis, and S. caprae (Table 3). C. indologenes, E. faecalis, and S. capitis also showed ≥4-fold increases in the MBCs, as did S. lugdunensis. Following 10 passages in the absence of PHMB, the MICs partially reverted to within a <2-fold difference from preexposure levels, with the exception of E. faecalis, which only partially reverted. Similarly, all test bacteria yielded X10 MBC values within a 2-fold difference from preexposure values, with the exception of C. indologenes (a 4-fold difference). Of note, B. cepacia, B. cereus, C. xerosis, and S. marcescens exhibited X10 MIC/MBC values that were moderately lower (≤3-fold) than those determined prior to microbicide exposure.

**TABLE 3:** MICs and minimum bactericidal concentrations of bacteria before and after treatment with polyhexamethylene biguanide^*a*^

Test bacterium	MIC (μg/ml)			MBC (μg/ml)		
	Before exposure	P10	X10	Before exposure	P10	X10
Bacillus cereus	58	29	58	58	58	58
Burkholderia cepacia	58	58	29	116	58	58
Chryseobacterium indologenes	0.9	**3.6**	1.8	1.8	**14.5**	**7.3**
Corynebacterium xerosis	2.7 (1)	7.3	2.2 (0.4)	21.8 (8)	7.3	14.5
Enterococcus faecalis	1.8	**14.5**	**9.7**	7.3	**29**	7.3
Escherichia coli	13.3 (3)	24.2 (8)	7.3	26.6 (6)	58	14.5
Klebsiella pneumoniae	7.3	**29**	9.7 (4)	29	96.7 (34)	58
Micrococcus luteus	1.8	**7.3**	1.8	7.3	14.5	7.3
Pseudomonas aeruginosa	31.3 (6)	58	29	116	232	116
Serratia marcescens	38.7 (15)	29	29	38.7 (15)	29	29
Staphylococcus aureus	7.3	7.3	7.3	52 (11)	58	58
Staphylococcus capitis	1.1 (0.3)	**6 (2)**	1.8	7.3	**48.3 (17)**	7.3
Staphylococcus caprae	6.7 (2)	4.9 (2)	7.3	29	38.7 (17)	29
Staphylococcus epidermidis	3 (1)	**14.5**	3.6	26.6 (6)	38.7 (17)	29
Staphylococcus haemolyticus	1.8	**7.3**	1.8	29	58	29
Staphylococcus lugdunensis	3.6	7.3	1.8	5.4 (2)	**48.3 (17)**	7.3
Staphylococcus warneri	3.6	6 (2)	3.6	29	58	29
Stenotrophomonas maltophilia	3 (1)	3.6	3.6	29	29	29

a See Table 1 footnote for explanation of data.

### Triclosan.

Eleven out of 18 test bacteria underwent a ≥4-fold increase in the MICs, and 9 out of 18 exhibited a ≥4-fold increase in the MBCs following 10 passages in the presence of triclosan (Table 4). The bacterium most susceptible to triclosan (MIC) was S. aureus. However, this bacterium also underwent the greatest reduction in susceptibility (MIC) following repeated sublethal microbicide exposure. In terms of the MBCs, E. coli was the most susceptible bacterium, followed by S. aureus and E. faecalis. Interestingly, these bacteria also exhibited the most pronounced changes in the MBCs during the investigation, with a 58-fold increase observed in the case of E. coli. P. aeruginosa was shown to be intrinsically nonsusceptible to all test concentrations of triclosan (27). After repeated cycles of growth in a triclosan-free medium, the MICs of the majority (8/11) of test bacteria reverted to preexposure levels with the exception of those for E. coli, K. pneumoniae, and S. aureus, which remained elevated. With regard to the MBCs, E. faecalis, E. coli, and S. aureus only partial reverted to preexposure values. The remaining test bacteria yielded MBCs comparable to preexposure levels following the cessation of triclosan dosing (X10) (Table 4).

**TABLE 4:** MICs and minimum bactericidal concentrations of bacteria before and after treatment with triclosan^*a*^

Test bacterium	MIC (μg/ml)			MBC (μg/ml)		
	Before exposure	P10	X10	Before exposure	P10	X10
Bacillus cereus	7.3	**29**	7.3	58	116	58
Burkholderia cepacia	232	116	232	464	464	464
Chryseobacterium indologenes	0.9	**3.6**	0.9	3.6	7.3	3.6
Corynebacterium xerosis	7.3	**58**	7.3	7.3	**58**	7.3
Enterococcus faecalis	3.3 (1)	**58**	3.3 (1)	3.3 (1)	**96.7 (34)**	**14.5**
Escherichia coli	0.5	**29**	**4.82**	0.5	**29**	**14.5**
Klebsiella pneumoniae	0.9	**116**	**14.5**	29	**116**	14.5
Micrococcus luteus	7.3	12.1 (4)	3.63	7.3	14.5	7.3
Pseudomonas aeruginosa	NS^*b*^	NS	NS	NS	NS	NS
Serratia marcescens	232	116	232	232	464	232
Staphylococcus aureus	0.2	**29**	**2.4**	1.8	**58**	**12.1 (4)**
Staphylococcus capitis	24.2 (8)	29	14.5	29	77.3 (33)	29
Staphylococcus caprae	12.3 (4)	29	14.5	24.2 (8)	58	29
Staphylococcus epidermidis	13.3(3)	**38.7 (17)**	14.5	53.2 (12)	116	58
Staphylococcus haemolyticus	0.4	**29**	0.4	7.3	**58**	7.3
Staphylococcus lugdunensis	0.9	**29**	0.9	7.3	**58**	7.3
Staphylococcus warneri	0.9	**24.2 (8)**	0.9	14.5	38.7 (17)	14.5
Stenotrophomonas maltophilia	14.5	**232**	14.5	58	**463**	48.3

a See Table 1 footnote for explanation of data.

b NS, nonsusceptible (MIC/MBC ratio of >1,000 μg/ml).

### ApoEdpL-W.

ApoEdpL-W was most potent against S. caprae and S. epidermidis, followed by S. warneri and C. indologenes. S. marcescens was the least susceptible of the test bacteria to the peptide (Table 5). With respect to changes in susceptibility following antimicrobial exposure, S. caprae exhibited the largest decrease in apoEdpL-W susceptibility after 10 passages, with the MIC value increasing by up to 21-fold (P10). After growth in peptide-free medium (X10), markedly increased MIC/MBC values (≥4-fold) partially reverted for a number of test bacteria, including C. indologenes, S. caprae, and K. pneumoniae. Changes in sensitivities (MBCs) for S. epidermidis and S. haemolyticus were stable, with no reversion in susceptibility being detected following 10 passages in the absence of the peptide.

**TABLE 5:** MICs and minimum bactericidal concentrations of bacteria before and after treatment with apoEdpL-W^*a*^

Test bacterium	MIC (μg/ml)			MBC (μg/ml)		
	Before exposure	P10	X10	Before exposure	P10	X10
Bacillus cereus	14.5	29	29	58	58	58
Burkholderia cepacia	29	29	29	58	58	58
Chryseobacterium indologenes	1.4 (0.4)	**14.5**	**3.63**	3 (1)	**14.5**	14.5
Corynebacterium xerosis	14.5	29	14.5	29	24.2 (8)	29
Enterococcus faecalis	7.3	**29**	**29**	7.3	**232**	**58**
Escherichia coli	58	29	29	58	96.7	29
Klebsiella pneumoniae	7.3	**29**	7.3	7.3	**29**	12.1 (4)
Micrococcus luteus	7.3	12.1 (4)	7.3	14.5	29	29
Pseudomonas aeruginosa	14.5	**48.3 (17)**	14.5	58	119.3	58
Serratia marcescens	232	464	232	464	464	464
Staphylococcus aureus	7.3	3.6	7.3	14.5	29	14.5
Staphylococcus capitis	13.3 (3)	24.2 (8)	14.5	29	9.8	29
Staphylococcus caprae	0.9	**19.3 (8)**	**3.6**	3.6	**29**	7.3
Staphylococcus epidermidis	0.9	**7.3**	**3.6**	4.2	**58**	**58**
Staphylococcus haemolyticus	1.3 (1)	**3.6**	**3.6**	3.6	**29**	**38.7 (8)**
Staphylococcus lugdunensis	7.3	3.6	1.2	7.3	7.3	7.3
Staphylococcus warneri	1.8	3.6	0.9	1.8	**19.3 (8)**	**14.5**
Stenotrophomonas maltophilia	14.5	7.3	14.5	14.5	14.5	14.5

a See Table 1 footnote for explanation of data.

### Changes in biofilm formation in antimicrobial-insusceptible microorganisms.

**FIG 1:**
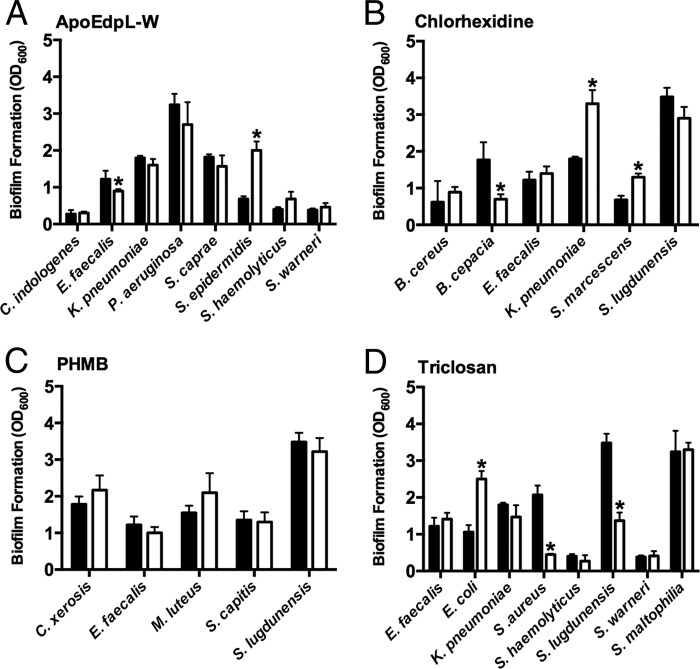
Bacterial biofilm formation before (■) and after (□) long-term exposure to ApoEdpL-W (A), chlorhexidine (B), PHMB (C), and triclosan (D). Data represent changes in biofilm formation in selected bacteria that underwent >4-fold changes in the MBCs following microbicide exposure. *, significant change in result (*P* < 0.001). Data show the mean levels of biofilm-bound crystal violet for P0 and P10 samples taken from two separate experiments each with three technical replicates.

Bacteria that had undergone >4-fold changes in the MBCs during the training procedure were further assessed for changes in their ability to form biofilms. The following bacteria exhibited a significant decrease in biofilm formation in a microtiter plate-based system: E. faecalis following exposure to apoEdpL-W, B. cepacia following exposure to chlorhexidine, and S. aureus and S. lugdunensis following exposure to triclosan. In contrast, repeated exposures of E. coli and S. epidermidis to triclosan and apoEdpL-W appeared to promote biofilm formation (Fig. 1), while chlorhexidine exposure was associated with increases in biofilm formation in K. pneumoniae and S. marcescens. For PHMB, no significant difference in biofilm formation was observed between the unexposed and exposed counterparts. In addition, none of the microorganisms investigated showed a >4-fold change in the MBC toward cetrimide, and they were therefore not examined for changes in biofilm formation.

## DISCUSSION

In agreement with previous *in vitro* studies (23, 24, 29, 34), repeated laboratory exposure of certain bacteria to microbicides resulted in decreases in bacterial susceptibility. Of the 34 ≥4-fold decreases in susceptibility (MICs) observed in this study, 15 were fully reversible, 13 were partially reversible, and 6 were nonreversible. Of the 26 ≥4-fold increases in the MBCs, 7 were fully reversible, 14 were partially reversible, and 5 were nonreversible. Readily reversible changes in susceptibility may result from temporary phenotypic adaptations, such as the induction of stress responses (35, 36) and changes in envelope composition (37) or efflux pump expression (22, 38, 39). In contrast, the reductions in antimicrobial susceptibility that were maintained after growth in the absence of the antimicrobial may be attributable to the selection of mutants (6).

While there are multiple reports in the literature of the laboratory generation of bacteria with decreased susceptibilities toward microbicides, adapted bacteria may remain effectively susceptible to in-use concentrations of the agent. For example, in the current investigation, the largest decrease in microbicide susceptibility occurred for S. aureus in response to triclosan, exhibiting a 45-fold increase in the MIC (0.2 μg/ml to 29 μg/ml) and a 32-fold increase in the MBC (1.8 μg/ml to 58 μg/ml) (Table 4); however, the in-use triclosan concentration, for example, in hand soaps is approximately 3,000 μg/ml, which is orders of magnitude higher than the observed elevated MIC and MBC (40, 41). Similarly, out of all the decreases in microbicide susceptibility observed in this investigation, no bacterium exhibited either an induced increase in the MIC/MBC or the wild-type MIC/MBC above in-use microbicide concentrations. The only bacterium which was nonsusceptible was P. aeruginosa toward triclosan; this intrinsic characteristic is not inducible and had previously been attributed to the expression of efflux pumps (41–43).

Bacterial susceptibility to antimicrobial agents can be markedly influenced by structural variations in the bacterial cell envelope which affect cell permeability (44–46). Barriers to microbicide cell penetration, such as the additional outer membrane in Gram-negative bacteria (47) or the presence of a spore coat in bacterial endospores (48), can confer protection against microbicides and possibly account for some of the low microbicide susceptibilities observed in these respective groups of bacteria in the current study. In Gram-positive bacteria, compounds such as QACs and biguanides may readily transverse the cell wall, making the bacteria relatively susceptible to these compounds (49, 50). However, as is apparent in the current investigation, susceptibility can range widely within each bacterial group. When challenged with a microbicide, a reduction in microbicide accumulation in the bacterial cell is a common survival mechanism and may be partly achieved by decreased cell permeability. The effectiveness of this strategy depends on several factors relating to the particular bacterium and the microbicide.

While triclosan induced the highest frequency and largest magnitude of changes in bacterial susceptibility and cetrimide exposure resulted in the lowest, all changes in the MICs and MBCs toward triclosan were either fully or partially reversible. The laboratory selection of bacteria with reduced susceptibility toward triclosan has been previously documented and has been attributed to mutations in the enoyl-acyl carrier reductase encoded by *fabI* or the overexpression of efflux pumps (6, 51). For example, reduced triclosan susceptibility in E. coli has been generated in the laboratory by the selection of bacteria with mutations in *fabI* or through the upregulation of the multidrug efflux pump AcrAB or its positive regulators, MarA and SoxS (51–53). Similarly, in another laboratory-based investigation, the exposure of S. maltophilia to triclosan selected for insusceptible variants that overexpress the SmeDEF multidrug efflux pump (39), while mutations in *fabI* in S. aureus have also been shown to reduce triclosan susceptibility (54).

The quaternary ammonium compound cetrimide and biguanides, such as PHMB and chlorhexidine, reportedly target the bacterial cytoplasmic membrane (1, 45, 47, 55) and the expression of multidrug resistance efflux pumps can influence bacterial susceptibility towards these agents (38; previously reviewed in reference 56). The plasmid-encoded OqxAB multidrug resistance pump reportedly conferred a decrease in cetrimide susceptibility in E. coli (57), and overexpression of the major facilitator superfamily efflux pump NorA in S. aureus has also been linked to reduced cetrimide susceptibility (58). In the present study, transient upregulation of efflux pumps may explain the decreases in the susceptibilities of E. coli, S. epidermidis, S. haemolyticus, and S. lugdunensis following cetrimide treatment. Furthermore, reductions in the permeability of the outer membrane have also been related to reduced microbicide susceptibility in many Gram-negative bacteria, particularly toward QACs (45) and biguanides (44). The mechanisms responsible include changes in lipopolysaccharide expression or structure (44), loss of porin proteins (59), and alterations in outer membrane fatty acid composition (45).

Efflux pump expression also apparently contributes to changes in biguanide susceptibility in bacteria (60). Fang and colleagues previously documented the isolation of chlorhexidine-nonsusceptible K. pneumoniae, which expressed a novel locus with a sequence compatible to that of a cation efflux pump, designated *cepA* (60). S. marcescens isolated from a chlorhexidine-containing contact lens solution exhibited alterations in outer membrane protein composition, which was linked to chlorhexidine nonsusceptibility (61). It is also possible that the induction of efflux mechanisms may have contributed to the reductions in biguanide susceptibility observed in the current study. Moore and colleagues previously examined the effect of sublethal PHMB exposure on a selection of bacteria isolated from the human skin and a domestic drain. Similar to our findings, they observed changes in the susceptibilities of various staphylococcal strains after PHMB exposure (23).

Four species of staphylococci exhibited decreases in apoEdpL-W susceptibility, three of which were nonreversible. It has been documented that staphylococci produce extracellular “V8” proteases that play a role in their pathogenesis (62). Certain cationic peptides are substrates for such proteases and, therefore, when expressed, confer stable cationic antimicrobial peptide (CAMP) resistance to the bacteria (62). Expression of efflux systems, such as the *qacA*-mediated efflux system in S. aureus, has also been associated with CAMP resistance in staphylococci. Furthermore, it has been shown that CAMP exposure in certain Gram-negative bacteria may induce protein, phospholipid, and lipopolysaccharide (LPS) modifications due to activation of the PhoP/PhoQ regulon (63), decreasing the attracting force between the positively charged peptide and negatively charged bacterial cell wall. In K. pneumoniae, a bacterial capsular polysaccharide (CPS) is thought to mediate CAMP resistance (64). K. pneumoniae was one of the few organisms that showed a widespread decrease in susceptibility to all the antimicrobials tested in this study. It is therefore plausible that upregulation of capsule synthesis in K. pneumoniae may confer a broad-range defense mechanism when antimicrobial stress is experienced.

As well as showing decreases in competitive fitness (65), bacteria adapted to grow in the presence of microbicides can display further phenotypic alterations such as decreases in growth rate, pigmentation, and biofilm formation, which could lead to altered pathogenic capability (30, 65, 66). After exposure to antimicrobials, several bacteria in the current study demonstrated significant alterations (increases and decreases) in their ability to form biofilms in a microtiter plate assay. The mechanisms responsible for such changes and their implications are currently poorly understood, but they may be due to the selection of mutants with alterations in factors directly involved in bacterial adhesion and biofilm maturation or the selection of isolates with altered growth rates and fitness, which can indirectly affect biofilm formation (30, 65). Any adaptation that renders a bacterium less susceptible to an antimicrobial may therefore also result in reduced or increased fitness (65, 67), which may influence pathogenic ability. An increased capacity to form biofilms was observed after apoEdpL-W adaptation in S. epidermidis. While the mechanisms underlying this change have not been elucidated, a similar effect observed for S. epidermidis after exposure to alcohol-containing skin disinfectants was explained on the basis of increased polysaccharide intracellular adhesin (PIA) synthesis (68). In the current study, S. marcescens and K. pneumoniae also exhibited increased biofilm-forming abilities after chlorhexidine exposure, which could potentially be mediated through altered capsule formation in K. pneumoniae (64, 69) or the upregulation of efflux pumps (60).

Decreases in bacterial specific growth rates have been reported following sublethal exposure to antimicrobials, and such changes may have influenced biofilm formation in our bacterial isolates (30, 70). The apparent decreases in biofilm formation observed for E. faecalis after apoEdpL-W exposure and B. cepacia after exposure to chlorhexidine may result from a lower density of cells within a slower growing culture, which could influence the expression of cell density-dependent genes involved in the process of biofilm formation (71). S. aureus and S. lugdunensis showed a decrease in biofilm formation after triclosan exposure. A decrease in staphylococcal biofilm production has previously been attributed to alterations in PIA and Agrocybe aegerita peroxidase (Aap) production or to changes in *sarA*, a regulatory gene which controls the expression of virulence determinants involved in biofilm development, such as DNase (72).

In conclusion, repeated exposure of bacteria to certain microbicides *in vitro* can result in decreases in antimicrobial susceptibility that may be transient or sustained, probably resulting from temporary phenotypic adaptations or the selection of stable genetic mutations, respectively. In adapting to antimicrobial stress, bacteria can exhibit alterations in other physiological characteristics, such as increases or decreases in biofilm-forming ability.
